# Daratumumab–bortezomib–dexamethasone use in relapsed POEMS syndrome

**DOI:** 10.1002/jha2.492

**Published:** 2022-06-05

**Authors:** Jahanzaib Khwaja, Ryan Keh, Duncan Smyth, Michael Peter Lunn, Shirley D'Sa, Jonathan Sive

**Affiliations:** ^1^ Department of Haematology University College London Hospitals London UK; ^2^ Centre for Neuromuscular Disease National Hospital for Neurology and Neurosurgery, Queen Square London UK

**Keywords:** Immunotherapy, multiple myeloma, myeloma, myeloma therapy

## Abstract

POEMS syndrome is a rareparaneoplastic disorder driven by an underlying low level plasma cell dyscrasiaand associated with elevated serum vascular endothelial growth factor (VEGF). Dueto its rarity, there are no internationally agreed standards of care, with verylimited data to guide management in the relapse setting. Agents used in myelomaare rational choices and have been employed. Daratumumab has been reported intwo case studies with lenalidomide‐dexamethasone, one in the upfront and one inthe relapsed setting. We are the first to report here three cases ofdaratumumab‐bortezomib‐dexamethasone (DVd) use in relapsed POEMS postautologous stem cell transplant with good VEGF and clinical responses. Our casesadd to the literature on efficacy of daratumumab and are the first to report onits safe use with bortezomib in relapsed POEMS. It should be considered as aclinical option, in patients not responding to conventional first linetherapies.

## 1 INTRODUCTION

POEMS syndrome (polyneuropathy, organomegaly, endocrinopathy, monoclonal gammopathy, skin changes) is a rare paraneoplastic disorder driven by an underlying low‐level plasma cell dyscrasia and associated with elevated serum vascular endothelial growth factor (VEGF). For patients with systemic presentation, treatment is aimed at eradicating the plasma cell clone. There is no internationally agreed standard of care, however induction chemotherapy followed by autologous stem cell transplantation (ASCT) is a favoured first‐line approach with proven excellent outcomes in large retrospective series [1, 2]. In the relapse setting, however, data to guide management are scant. Agents used in myeloma are rational choices and have been employed. Daratumumab has been reported in two case studies with lenalidomide–dexamethasone (Rd), one in the upfront [3] and one in the relapsed setting [4]. We report here our first three cases of daratumumab–bortezomib–dexamethasone (DVd) use at our centre, with ongoing VEGF and clinical responses.

### 1.1 Case 1

A 51‐year‐old female presented with fatigue, abdominal bloating and paraesthesia of the lower limbs and hands. She had an IgA lambda monoclonal protein (M‐protein) of 2 g/L with no abnormal infiltrate on bone marrow biopsy. Whole body computed tomography (CT) scan showed generalised lymphadenopathy, moderate pericardial effusion and splenomegaly at 18 cm. A diagnosis of multicentric Castleman's disease was made from lymph node biopsy. Despite administration of four cycles of Rituximab monotherapy, no symptomatic or objective imaging benefit was derived. On reassessment, serum VEGF was raised at 2960 pg/ml, nerve conduction studies showed a sensorimotor polyneuropathy and a diagnosis of POEMS syndrome was made.

Eight cycles of Rd (lenalidomide 15 mg days 1–21, dexamethasone 20 mg days 1, 8, 15, 22) were delivered. After an initial clinical response, VEGF began to increase again to 1565 pg/ml. She had a melphalan (140 mg/m^2^) conditioned ASCT. Despite achieving a complete VEGF response, by day 100 she clinically deteriorated with refractory ascites requiring drainage, pleural effusions and persistent severe thrombocytopenia and neutropenia. Repeat bone marrow demonstrated relapse (10%–20% neoplastic plasma cell infiltrate), M‐protein 1 g/L, but no FDG avid lesions on CT‐positron emission tomography (PET) scan.

Because of clinical progression, second line DVd (daratumumab 1800 mg subcutaneous, bortezomib 1.3 mg/m^2^ subcutaneous days 1, 8, 15, 22; dexamethasone 20 mg) was started. Daratumumab was given weekly for 9 weeks, every 3 weeks from weeks 10–24 followed by every 4 weeks from week 25 onwards. By eight months, the ascitic drain was removed without fluid accumulation, peripheral oedema and pleural effusions had resolved and she had improvement in cytopenias. She tolerated treatment well without intercurrent infections or bortezomib‐associated neuropathy. M‐protein is undetectable, with complete VEGF and PET responses, as well as clinical improvement. She remains on daratumumab maintenance (cycle 16).

### 1.2 Case 2

A 31‐year‐old male presented with cervical lymphadenopathy and parotid swelling. Parotid histopathology was consistent with Castleman's disease and staging CT scan showed sclerotic bone lesions. After a year of observation, he developed drenching night sweats and progressive lower limb sensory neuropathy. A monoclonal IgG lambda M‐protein of 11 g/L was found with no plasma cell infiltrate on bone marrow trephine. Lambda light chain restricted plasma cells were present in a pelvic bone biopsy and VEGF 1102 pg/ml, leading to a diagnosis of POEMS syndrome. He underwent upfront melphalan‐conditioned (200 mg/m^2^) ASCT with complete VEGF and PET response, M‐protein was undetectable and clinical symptoms improved.

Five years later, he relapsed with headaches, presyncope, foot drop and erectile dysfunction. Cerebral magnetic resonance venogram confirmed an acute venous sinus thrombosis, whole body CT‐PET revealed multifocal new high‐grade avidity in skeletal lesions with a large iliac lesion and extraosseus soft tissue extension. VEGF had risen to 9045 pg/ml, IgG M‐protein to 15 g/L and bone marrow trephine showed 10% plasma cell infiltrate. Second‐line DVd (daratumumab 1800 mg subcutaneous, bortezomib 1.3 mg/m^2^, dexamethasone 20 mg) was commenced alongside radiotherapy to left pelvis (20 Gy in five fractions). He tolerated treatment well without worsening of neuropathy with a complete VEGF response by 2 months, partial haematological response by 4 months, partial PET response and overall good clinical response. After 28 cycles of DVd, he remains in remission and continues maintenance daratumumab. He has experienced neither bortezomib‐associated neuropathy nor infections.

### 1.3 Case 3

A 58‐year‐old male presented with progressive leg oedema, weight loss, widespread glomeruloid haemangiomas over the trunk and limbs and bilateral leg weakness requiring wheelchair use. Whole body CT showed widespread sclerotic bone lesions; an IgA lambda M‐protein 3 g/L was found with 25% plasma cell infiltration on bone marrow and VEGF of 8030 pg/ml. Despite four cycles of Rd followed by melphalan ASCT (140 mg/m^2^) with a partial haematological (post‐ASCT marrow infiltration 5%) and initial VEGF response (nadir 2910 pg/ml 3 months post‐ASCT), he quickly relapsed. He therefore commenced second‐line DVd 6‐month post‐ASCT. He tolerated this well and has now completed three cycles. He has had clinical improvement with weight, reduced leg swelling and VEGF reduced from 6605 to 2855 pg/ml, which is the lowest he has ever recorded and M‐protein undetectable.

## 2 DISCUSSION

Historically, there have been concerns of neurotoxicity with bortezomib which have led to its reduced use, however studies support its safety in POEMS. A study of 20 patients who received biweekly intravenous bortezomib in combination with dexamethasone and cyclophosphamide had no neurological toxicity [5]. In the myeloma context, bortezomib has been shown to have equal efficacy on a weekly rather than biweekly schedule and subcutaneous rather than intravenous with additional reduced rates of peripheral neuropathy [6, 7]. The largest series of bortezomib–dexamethasone use in POEMS recently reported on 69 patients at 1.3 mg/m^2^ subcutaneously [8] with excellent outcomes (haematological complete remission rate 46%, VEGF complete remission rate 71%, neurological improvement 88%, 2‐year time to next treatment 66%) and only 3% developed grade 1 bortezomib‐induced neuropathy after six cycles of treatment, which was reversible after drug withdrawal. A multicentre study comparing upfront therapies showed that bortezomib was the most efficacious treatment (69% haematological complete remission/very good partial response compared with immunomodulatory drugs and alkylators) with no treatment discontinuation [9].

There is now substantial evidence for the additional benefit of anti‐CD38 monoclonal antibody treatment when added to anti‐myeloma therapy from multiple trials in the frontline (MAIA, CASSIOPEIA, GRIFFIN) [10, 11, 12] and relapsed settings (CASTOR and POLLUX) [13, 14]. In the context of bortezomib‐containing regimens, the CASTOR study demonstrated improved progression‐free survival in patients with relapse/refractory myeloma with the addition of daratumumab to bortezomib–dexamethasone [13]. Given that much of the treatment paradigm for POEMS syndrome is taken from myeloma, it is reasonable to use daratumumab in this context too.

**FIGURE 1:**
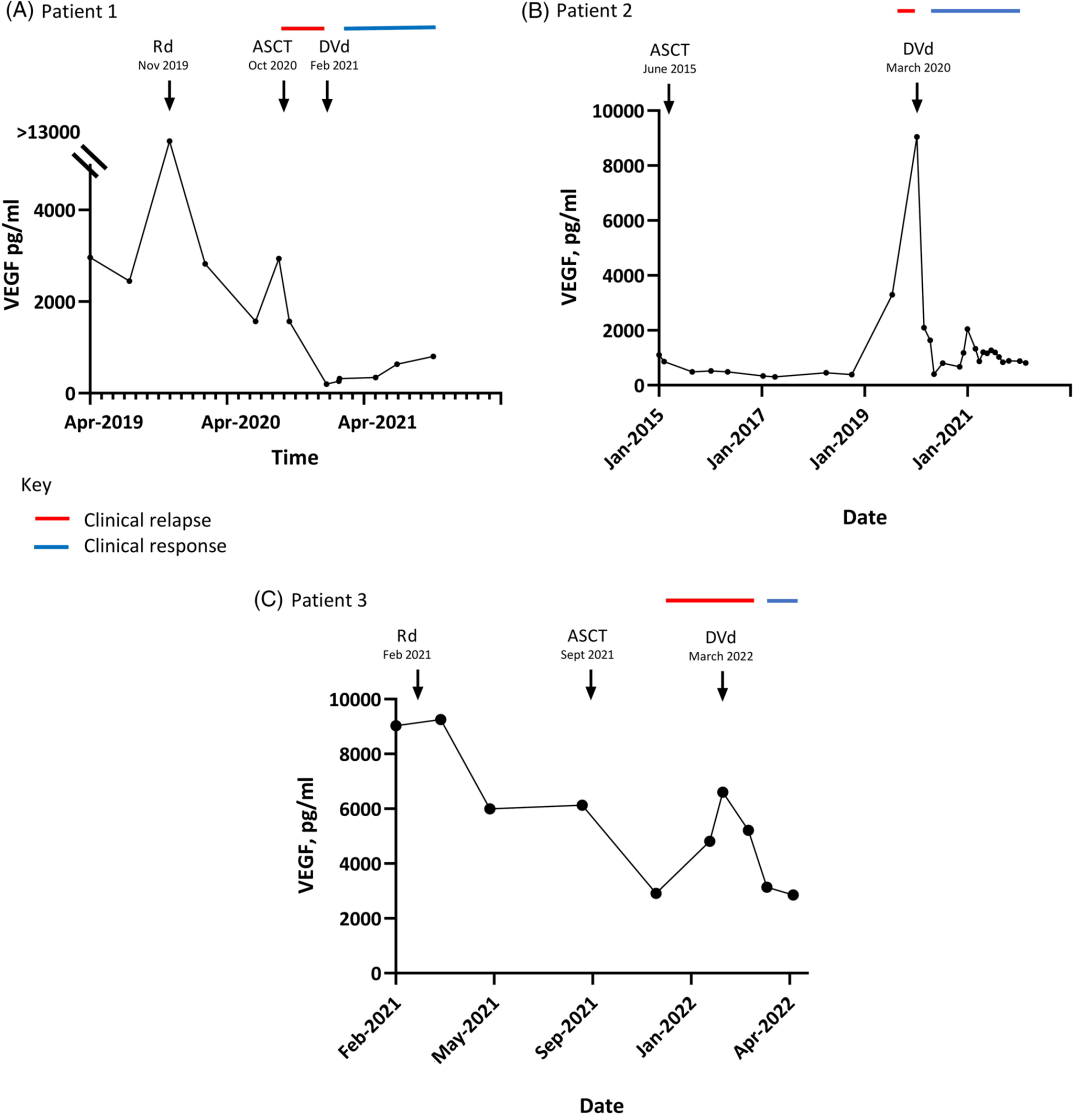
Patient vascular endothelial growth factor (VEGF) and clinical responses

Our cases add to the literature on efficacy of daratumumab and are the first to report on its safe use with bortezomib in relapsed POEMS. Daratumumab should be considered as a clinical option in patients not responding to conventional first line therapies (see Figure 1).

## CONFLICT OF INTEREST

Jahanzaib Khwaja, Ryan Keh, Duncan Smyth, Michael Peter Lunn, Jonathan Sive declare no conflicts of interest; Shirley D'Sa reports speaker fees and research funding from *Janssen*, *BeiGene* and *Sanofi*.

## FUNDING INFORMATION

The authors received no specific funding for this work.

## PATIENT CONSENT

Consent for publication was obtained from all patients.

## PERMISSION TO REPRODUCE MATERIAL FROM OTHER SOURCES

No excerpts from copyrighted works are included

Daratumumab‐bortezomib‐dexamethasone use in relapsed POEMS syndrome

## Data Availability

The data that support the findings of this study are available on request from the corresponding author. The data are not publicly available due to privacy or ethical restrictions.
